# Sensor-Fusion Based Navigation for Autonomous Mobile Robot

**DOI:** 10.3390/s25041248

**Published:** 2025-02-18

**Authors:** Vygantas Ušinskis, Michał Nowicki, Andrius Dzedzickis, Vytautas Bučinskas

**Affiliations:** Department of Mechatronics, Robotics and Digital Manufacturing, Faculty of Mechanics, Vilnius Gediminas Technical University, LT-10105 Vilnius, Lithuania; vygantas.usinskis@vilniustech.lt (V.U.); michal.nowicki@vilniustech.lt (M.N.); andrius.dzedzickis@vilniustech.lt (A.D.)

**Keywords:** sensor fusion, mobile robot, navigation, machine learning

## Abstract

Navigation systems are developing rapidly; nevertheless, tasks are becoming more complex, significantly increasing the number of challenges for robotic systems. Navigation can be separated into global and local navigation. While global navigation works according to predefined data about the environment, local navigation uses sensory data to dynamically react and adjust the trajectory. Tasks are becoming more complex with the addition of dynamic obstacles, multiple robots, or, in some cases, inspection of places that are not physically reachable by humans. Cognitive tasks require not only detecting an object but also evaluating it without direct recognition. For this purpose, sensor fusion methods are employed. However, sensors of different physical nature sometimes cannot directly extract required information. As a result, AI methods are becoming increasingly popular for evaluating acquired information and for controlling and generating robot trajectories. In this work, a review of sensors for mobile robot localization is presented by comparing them and listing advantages and disadvantages of their combinations. Also, integration with path-planning methods is looked into. Moreover, sensor fusion methods are analyzed and evaluated. Furthermore, a concept for channel robot navigation, designed based on the research literature, is presented. Lastly, discussion and conclusions are drawn.

## 1. Introduction

In the rapid development of the automation and robotics world, making of autonomous vehicles and mobile robots is a big step toward operational efficiency, safety, and autonomy. At the heart of this technological revolution is the intricate domain of sensor fusion, a paradigm that merges data from different sensors to make cohesive and accurate perceptions of operational environments. This paper goes into the realm of sensor-fusion-based navigation systems for autonomous robots, spotlighting diverse methodologies that underpin their functionality and emerging trends that shape their evolution.

Navigational autonomy in robots is paramount for their effective deployment across a spectrum of applications, from industrial automation to exploration in inaccessible terrains. Traditional navigation methodologies, while foundational, often grapple with complexities and dynamic changes intrinsic to real-world environments. Bridging this gap, advanced navigation systems harness the synergy of global and local navigation methods [1]. Global navigation operates on the premise of pre-acquired environmental knowledge, facilitating formulation and adherence to predetermined paths. In contrast, local navigation equips mobile robots with agility to dynamically refine their paths in real time, utilizing an arsenal of external sensors—ranging from infrared and ultrasonic sensors to LASER, LIDAR, and cameras [2]. This sensorial diversity, when orchestrated by sophisticated software algorithms, enables autonomous correction of robot orientation and trajectory, ensuring navigational resilience against unforeseen obstacles and alterations in environment [3].

The dichotomy of global and local navigation methods embodies methodological diversity in robotic navigation, allowing robots to chart optimal paths and fulfil their designated tasks within varied environmental contexts. Nevertheless, reliance on prior environmental knowledge or capability for real-time path adjustment underscores limitations of classic navigation approaches [4,5]. These systems often operate within a deterministic framework, wherein navigation paths are predetermined, or a non-deterministic framework that allows for probabilistic path planning based on sensor input and environmental interaction [6].

A non-deterministic framework becomes very relevant in applications that require navigation in hazardous and physically difficult to reach places for humans—for example, inspection of narrow underground channels. That kind of working environment lacks global reference points that could be used for a deterministic framework. Furthermore, there is the probability of encountering unexpected obstacles. For these reasons, integration of sensors for robot localization is a must.

Amidst these methodologies, optical data-based localization emerges as a critical area of focus, leveraging visual information to enhance a robot’s environmental awareness and decision-making capability. However, reliance on optical data introduces unique challenges for navigation, including the need for sophisticated object recognition algorithms and the ability to define navigational paths without explicit recognition cues [7,8].

As we delve deeper into the big picture of research in sensor-fusion-based navigation, this paper aims to elucidate myriad localization methods that empower mobile robots to traverse and interact with their surroundings effectively. By analyzing limitations of classic localization approaches and addressing challenges posed by optical data reliance, we seek to highlight the transformative potential of sensor fusion in crafting more adaptable, reliable, and sophisticated autonomous navigation solutions primarily focused on local path planning.

In anthropocentric terms, localization methods can be classified into vision-based and non-vision-based approaches, which makes the distinction easier to grasp. Vision-based methods rely on imaging cameras to capture visual information, similar to human sight, which is then analyzed in various ways to understand and navigate the environment. Non-vision-based methods, in contrast, use sensors like LIDAR, radar, ultrasonic etc., which perceive the environment through means that are alien to human senses, such as detecting distances through sound waves or localizing oneself through RFID tags.

The manuscript is organized to provide a comprehensive review of sensor-fusion-based navigation systems. Section 2: A literature search method details systematic processes, databases, and inclusion criteria used to gather relevant studies. Section 3: Navigation methods review global and local approaches, discussing their principles, strengths, and limitations. Section 4: Analysis of non-vision-based localization systems highlights technologies like ultrasonic, infrared, LiDAR, and radar sensors, while Section 5: Analysis of vision-based localization systems examines both standalone and hybrid configurations, focusing on integration and challenges. Section 6: Essential sensor fusion systems classify fusion architectures into cooperative, complementary, and competitive approaches, exploring key methodologies. Section 7: A solution for channel robot navigation presents exemplary cost efficient sensor fusion based local navigation system intended for mobile robots functioning in channels that cannot be physically reached by a human, combining RGB cameras, laser pointers, and pseudo-LiDAR. Finally, Section 8: Discussion and conclusions summarize key findings, emerging trends, and future directions in sensor fusion for robotics. 

## 2. Literature Search Method

The literature search method was based on the systemic process presented in article [9], which focuses on preferred reporting items of systematic reviews and meta-analyses (PRISMA) statement. Four main databases were utilized, including MDPI, IEEE Xplore, Google Scholar, and Science direct. Other specific databases were also used if there was no other way to access a required paper. The main criteria focusing on autonomous robot navigation topic were formed for the inclusion in this survey, such as:

- Focused on sensor application
- Focused on path planning
- Focused on mapping techniques
- Focused on sensor fusion method adaptions
- Focused on machine learning adaptions

Additional criteria for narrowing the main topic:

- Articles that are older than 5 years were excluded with some exceptions if specific points needed more investigation.
- Articles that do not focus on mobile robot navigation were excluded except if specific technology being investigated needed more input.
- Articles focusing on railways and sea navigation were not taken into consideration with the exception of several articles presenting air navigation systems.

**Figure 1:**
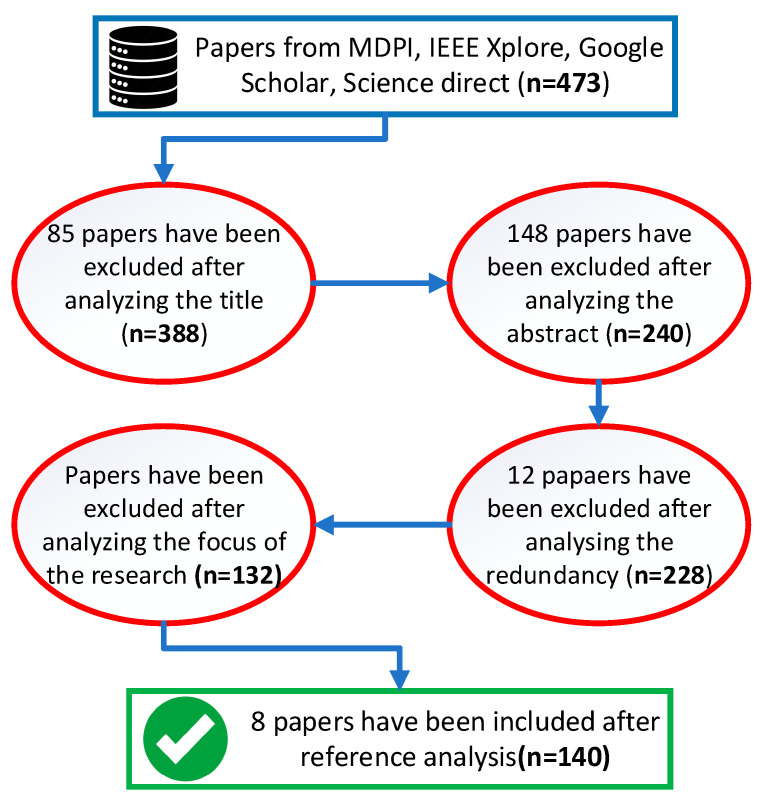
A systematic literature review workflow.

The main keywords that were selected for research on sensor fusion and autonomous mobile robot included in this manuscript were: “Sensor fusion”, “YOLO”, “Mobile robot”, “Kalman filter”, “Sensors for navigation”, “Path planning methods”, “LiDAR and camera fusion”, and “ML based sensor fusion”. A simplified workflow of the concluded survey for this manuscript is shown in Figure 1.

## 3. Navigation Methods

In global navigation, knowing the environment beforehand is base for making complete paths from start to end. This method needs a detailed map of the terrain, where the robot’s journey is decided by the environmental map it has. Some of the most popular path planning methods for global navigation are shown in Table 1. The challenge here is for the robot to match its planned path with real situations it meets, which is made harder by dynamic changes in the environment or if the global target point cannot be accurately established because of obstructions.

On other side, local navigation relies on robot’s ability to adjust in moment, using different external sensors for making decisions on the go. From the accuracy of LASER and LIDAR to depth seeing by cameras, these sensors are the robot’s eyes and ears, letting it see and react to obstacles with agility. Software algorithms work like a conductor in this, mixing data to guide the robot’s moves every moment. Some of the most popular path planning methods for local navigation are shown in Table 1.

Merging global and local navigation shows a mixed way, where a robot is given a wide environmental model but also keeps flexibility to change as needed. This mix improves the robot’s wayfinding, giving it paths that are both planned and reactive.

Sensor fusion stands as a key part in evolving navigation systems, bringing together different data streams into one clear understanding of surroundings. By putting together strengths of various sensors, from wide views of LIDAR to detailed capture by cameras, robots obtain a fuller view of their surroundings. This richer sensing not only makes path planning better but also helps robots move through complex, unstructured places.

**Figure 2:**
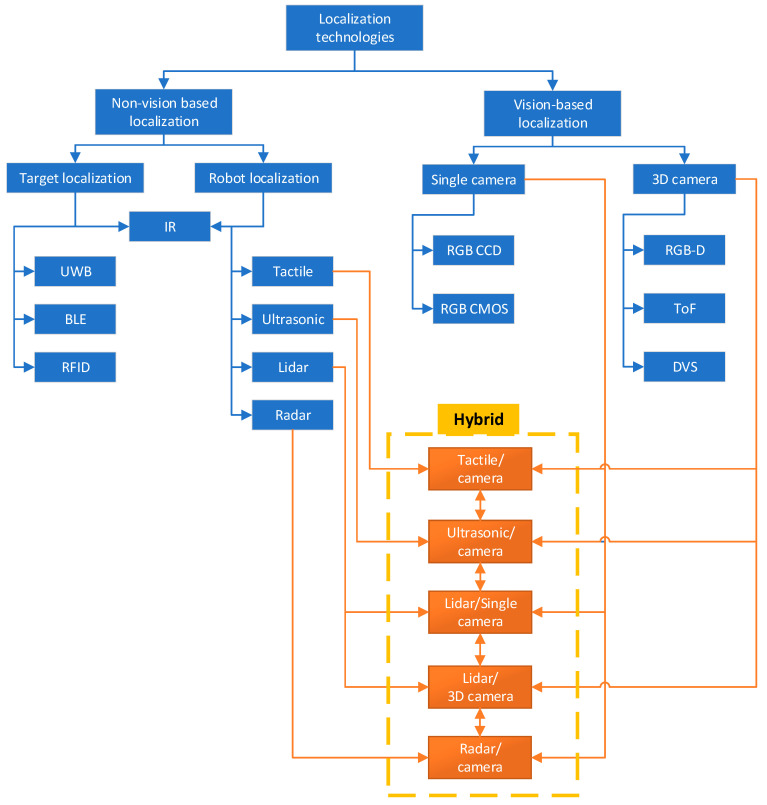
Summarized common hybrid sensor combinations for mobile robots.

But the path of innovation in robot path planning is ongoing, with new explorations and improvements always on horizon. Moving forward, bringing in new techs, with advances in machine learning and artificial intelligence, opens new possibilities in autonomous navigation. Bringing together global and local methods, backed by the power of sensor fusion, points to a future where robots move with unmatched precision, efficiency, and autonomy. Some of the technologies widely used for autonomous robot localization are shown in Figure 2.

## 4. Analysis of Non-Vision-Based Localization Systems

Non-vision-based localization technologies play a crucial role in the field of robotics, especially in environments where visual data may be unreliable or unavailable. These technologies encompass a variety of methods and sensors designed to enhance a robot’s ability to localize and navigate itself within its environment, leveraging alternative sensory data to achieve precise and reliable navigation. The same is true of the common non-vision-based technologies, which are shown on the left side of Figure 2.

One significant branch of non-vision-based localization focuses on target localization. This involves determining the position of specific targets within an environment, utilizing technologies such as Ultra-Wideband (UWB), Bluetooth Low Energy (BLE), and Radio-Frequency Identification (RFID). UWB technology, known for its high accuracy and reliability, is widely used in indoor positioning systems due to its ability to provide precise location information even in complex environments [21]. BLE, on the other hand, is commonly employed for proximity detection and location tracking, benefiting from its low power consumption and widespread use in consumer electronics [22]. RFID systems offer another layer of versatility, allowing for the identification and tracking of objects through electromagnetic fields [23]. These technologies collectively enhance the ability of robots to locate and interact with various targets, crucial for applications such as inventory management and asset tracking.

Robot localization, another critical aspect of non-vision-based localization, involves methods that enable robots to determine their own position within an environment. Infrared (IR) sensors are versatile tools used in both target and robot localization, providing reliable distance measurements and object detection capabilities [24]. Tactile sensors, which detect physical contact with objects, are particularly useful in cluttered environments where precise positioning is essential [25]. Ultrasonic sensors, employing sound waves to measure distances, are effective for obstacle detection and navigation in various conditions, including occluded vision due to fog or smoke or underwater environments [26]. Lidar (Light Detection and Ranging) systems stand out due to their ability to create high-resolution maps of the environment using laser pulses, offering unparalleled accuracy and detail [27,28]. Radar systems, which use radio waves, provide robust performance in diverse environmental conditions, making them indispensable for applications requiring reliable distance, angle, and velocity measurements [29]. To unravel and compare non-vision sensors for robot localization, methods proposed in the literature were analyzed and presented in Table 2.

From Table 2, we can see a variety of solutions to effectively achieve local navigation by incorporating proximity and contact sensors of different physical nature to detect obstacles. Due to field view limitations, it is noticeable that ultrasonic and IR distance sensors are usually used in combinations to compensate for those disadvantages. LiDAR and radar sensors have higher accuracy and field of view but require more efficient mapping techniques to increase performance. Further comparison of analyzed sensors is shown in Table 3.

As shown in Table 3, tactile sensors computationally lack proximity evaluation capabilities but are very computationally efficient. They are a great addition not only for obstacle detection purposes but also for collaborative function with human operators. Also, it is worth mentioning that in recent studies, tactile sensors vary in complexity and can even become a system of several sensors to measure contact and deformation phenomena. For example, in article [46], an optical tactile sensing system is presented, which can measure force distribution for arial mobile robot purposes.

The integration of these non-vision-based navigation technologies into robotic systems addresses several challenges associated with visual data reliance. For instance, varying lighting conditions and the need for sophisticated object recognition algorithms can complicate vision-based navigation. Non-vision-based systems, leveraging a combination of sensory inputs such as IR, tactile, ultrasonic, lidar, and radar, can navigate and localize effectively without the constraints of visual data. This adaptability is particularly advantageous in environments like warehouses, underwater explorations, and subterranean locales such as mines or tunnels where visual cues are limited or non-existent.

## 5. Analysis of Vision-Based Localization Systems

### 5.1. Standalone Vision Navigation Systems

Vision capability is an essential feature for mobile robot navigation systems. Many cameras were proven to work in this scenario with corresponding advantages and disadvantages, some of which are shown one the right side of Figure 2.

Camera devices can be separated into single and 3D cameras. Single camera can take 2D images. Most commonly used single cameras frequently used in robotic systems are RGB cameras based on CCD or CMOS sensors, which represent each taken pixel in an extensive spectrum of colors extracted from red, green, and blue color space [47]. They are highly applied for navigation. In article [48], an RGB camera is used to detect road lines according to the color so vehicles could follow the path in combination with other sensors. Other notable examples of single cameras are NIR cameras, which are less sensitive to visible light, meaning images are not corrupted by reflections [49]. Also, a fisheye camera is a powerful omni-directional perception sensor. It is used in navigation systems because of its wide field of view. In article [50], a fisheye camera is used to take images from 180 angle using the ASIFT algorithm to extract features of obstacles. Another edition for visions devices is the polarized camera, which had polarization systems able to extract orientation of the light oscillations reflected from perceived surfaces [51]. It is very convenient for detecting objects in crowded environments by filtering unwanted reflections and glare and enhancing the image contrast.

Depth measurement capability allows not only color recognition but also evaluation of object 3D geometry. One of the most frequently used cameras for this purpose is the RGB-D camera, which emits a predefined pattern of infrared light rays and the depth of each pixel is calculated by the reflection of rays [52]. Similarly, time of flight IR cameras work by illuminating present objects with modulated light and observing reflections, allowing the robot to perceive depth [53], although color cannot be perceived with this camera. Another increasing in popularity is the event-based camera, frequently employing DVS sensors, which capture pixel intensity changes, and robust compared to other cameras [54]. These cameras can also calculate depth by event capture, although it is computationally demanding and methods for efficiency are needed.

All mentioned cameras have corresponding advantages and disadvantages. To evaluate their properties and functionality, some of the researched methods for mobile robots and other types of navigation that integrate cameras in their systems will be analyzed. The researched methods are presented in Table 4.

**Figure 3:**
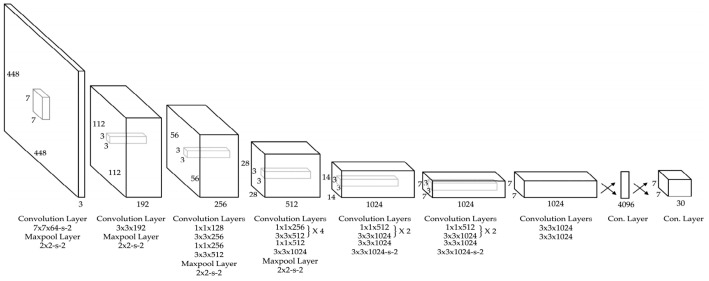
Typical YOLO network architecture consisting of 24 convolutional layers [63].

From Table 4, we can see a wide application of cameras for navigation purposes. Several techniques to effectively use vision devices for recognition were mentioned. One of the most popular techniques improving rapidly is you only look once (YOLO) and its advanced versions, which can work with high accuracy and speed in real time. It converts a target detection problem into a regression problem, dividing images in grids and making predictions for each grid cell separately [61]. YOLO incorporates convolutional neural network (CNN) principles to train and predict image data [62]. A typical YOLO network architecture is shown in Figure 3.

The first 24 convolutional layers extract features from the image, and the two fully connected layers predict the output bounding boxes and class probabilities directly from image pixels. Models from YOLO-v1 to the newly developed YOLO-v9 improved significantly. Going from YOLO-v1 to YOLO-v8 increased processing speed from 45 to 280 FPS and increased detection accuracy of 53.9% [64]. As stated in article [65] newly developed YOLO-v9 further increases detection accuracy by reducing information loss, which is encountered in sequential feature extraction process by utilization of programmable gradient information.

To select the most effective visions system for a specific project, it is important to know not only image recognition methods but also properties of devices. From the research papers, the properties of the most used cameras were summed up for comparison in Table 5.

From Table 5, it can be seen that CCD and CMOS cameras are the most cost efficient and have established methods for efficient object recognition tasks. They lack depth capability compared to other cameras in the table. Nevertheless, if it is convenient for a project because of the advantages mentioned, it is possible to measure depth with these cameras to a certain accuracy. For example, in a previously mentioned article [57], the triangulation principle was used to detect changes in lase pointer projection to estimate distance. Also, using similar triangulation principles, two cameras positioned at slightly different positions can measure depth by matching taken images [78]. By measuring the required time for reflected light to go from the source and come back, the concept of ToF sensors is designed. ToF cameras working in an infrared range are very convenient for accurate depth estimation. On the other hand, RGB-D can not only estimate depth based on similar principles but also detect a wide range of colors, but it is moderately more expensive than previous cameras and requires smarter algorithms for more efficient matching of color and depth. For example, in article [79], adaptive color-depth matching is proposed using a transformer-based framework to enhance computational performance. Lastly, event-based cameras enhance capabilities of object detection even more with high dynamic range and depth measuring capabilities. Nevertheless, these cameras are more expensive and challenge current AI-based methods for more effective performance.

### 5.2. Hybrid Visions Localization Systems

As previously explained, depth and field of view estimation with cameras is limited and, in some cases, expensive. Moreover, certain surfaces introduce challenges for detection. For this reason, in robotic navigation systems, cameras are commonly integrated in combination with other distance measurement sensors to enhance overall perception of working environments. Some of the common fusion combinations are shown in Figure 2.

As navigation environments are becoming more complex with dynamic obstacles and crowded spaces, infrastructures having more than one sensor became the staple of localization, combining sensors that can detect different physical phenomena. To obtain a better understanding of the advantages and disadvantages of hybrid systems, proposed methods in the literature were analyzed. Some of the methods are shown in Table 6.

Going through analyzed approaches of hybrid sensor methods in Table 6, it is clear that richer data can be acquired from working environments. Combining distance and visual sensors enables significantly more accurate object detection, which is achieved by mapping accurate distance data with visual data. On the other hand, all presented methods deal with high computational resources. To increase performance of mapping sensor data, several methods were established in time. One of the most widely used is simultaneous localization and mapping (SLAM), which utilizes data from the camera, distance, and other sensors and concurrently estimates sensor poses to generate a comprehensive 3D representation of the surrounding environments [86]. LiDAR and visual SLAM are well-known techniques, but the need to fuse different sensors established new algorithms. For example, in article [87], LiDAR inertial camera SLAM is proposed, enabling accurate tracking and photorealistic map reconstruction using 3D Gaussian splatting.

The core of hybrid sensors systems are fusion methods including Kalman filters, particle filters, and AI methods, which drastically affect the performance of the system. These methods will be introduced further in the next chapter. It is also important to choose the right devices for the project according to sensor properties, which affect the overall performance of the system. A comparison between different hybrid sensors combinations is presented in Table 7.

From Table 7, it can be seen that depth capabilities of RGB, RGB-D, and DVS cameras are enhancing significantly in fusion with distance sensors. To achieve the highest accuracy, DVS and Lidar solutions show a lot of promise, because DVS cameras also have low sensitivity to disturbances. If cost-efficient solutions with range capabilities are needed, then combining ultrasonic or radar sensors with cameras is a way to go. Combination of tactile sensors with cameras might not provide range but can be used for force-sensitive applications to detect and inspect object geometry and even material properties.

## 6. Essential Sensor Fusion Systems

Sensor fusion is an essential part of navigation because standalone systems based on one or two sensors cannot cope with increasing complexity of working environments and required tasks. As mentioned before, the addition of cooperating or competitive sensors allows an increase in the overall properties of the system including field of view and accuracy, taking into account different physical phenomena to generate better understanding about working environments and internal processes. To maximize the performance of sensor fusion, it is important to choose appropriate architecture depending on required tasks and chosen sensors. For better understanding, sensor fusion is classified by several factors in the literature. One of the main factors that regularly appears in the literature [100,101] defines how early sensor data are interconnected during data processing steps. It can be interpreted as abstraction level. Sensor fusion level according to abstraction can be classified as:

- Low-level—indicates that raw sensor data are directly sent to fusion module. This way no data are lost because of noise introduced by postprocessing, meaning some relevant data would not be overlooked. For example, in article [102], LiDAR 3D point cloud points are augmented by semantically strong image features significantly increasing the number of detected 3D bounding boxes. Nevertheless, high computational resources are required to compute raw data. Also, fusion modules are less adaptive because adding new sensors requires adjustments to the new sensor format.
- Medium-level (Feature)—involves extracting some key features from raw sensors. Due to this, bandwidth is reduced before carrying data fusion and similar efficiency of extracting relevant data is achieved. Also, this structure is more adaptive and adjustable. This is a very commonly used fusion method, then optimization is important. For instance, in article [103], encoder, color image, and depth image are first pre-processed before fusion. Unnecessary noise is removed from images to filter only required regions, and encoder provides orientation, ultimately creating a system capable of object recognition and robot localization.
- High-level—according to this structure, each sensor is postprocessed and carries out its task independently, and then high-level fusion of detected objects or trajectories by each sensor is performed. This type of fusion has high modularity and simplicity. On the other hand, key sensor data at lower levels are lost.

Another way to classify sensor fusion architectures is by relationship among the sources as listed in article [104], separating into three groups:

- Complementary—sensor information does not directly depend on one another but then combined can provide a more complete picture of observed phenomena.
- Competitive (redundant)—same or similar information is received from sensors to reduce uncertainties and errors, which could appear if using sensors separately.
- Cooperative—involves combined information extraction that cannot be acquired using one sensor. Involves active sensor collaboration exchanging insight and or intermediate data and increasing accuracy and reliability of overall fusion system.

To design proposed architectures and realize sensor fusion, specific methods and algorithms are required including Kalman, particle filters, novel neural network approaches, etc. To obtain a better understanding of sensor fusion architectures and methods used for mobile robot navigation and classification tasks, proposed solutions in literature are analyzed and presented in in Table 8, Table 9 and Table 10 below.

Low-level fusion architecture is useful for systems that require maximizing acquired data from the sensors with no loss for higher accuracy. Systems presented in the Table are designed for obstacle and human detection tracking tasks. These tasks must be performed with upmost accuracy to ensure safety in for all elements in the working environment. To integrate low-level fusion architecture in modern systems, which require real-time capabilities and communication between various software and hardware elements, optimization is necessary to reduce computational load.

High-level sensor fusion requires significant computing resources, and often these facilities are located remotely and connected via a fast network; therefore, known realized cases are less numerous except the previous ones.

Comparing analyzed sensor fusion approaches, it can be seen that for mobile robot navigation systems, which mainly focus on robot and target localization, cooperative mid-level sensor fusion architectures are dominant. Navigation requires not only accuracy but also efficiency to perform localization tasks faster. Due to this, mid-level sensor fusion architectures are convenient. Nevertheless, the system has to evaluate more phenomena which are not directly dependent on one another, and complementary fusion becomes handy. This is especially common in vehicle-to-everything communication. For example, in article [119], high-level fusion structure is presented where LiDAR and Radar is tasked with distance measurement and obstacle detection, and the camera complements the system by classification of objects. There are also plenty of modular-type sensor fusion architectures in autonomous robotic systems. For example, in article [120], a human detection system is designed with complementary sensor fusion. There, LiDAR is used to detect the lower part of a human and camera for the upper part of pose recognition.

To realize the designed structure of sensor fusion, the next step is to choose appropriate methods and algorithms to interconnect sensor data for correct estimation of system state. Going through the analyzed approaches in Table 9, several methods can be distinguished, which will be presented below.

### 6.1. Sensor Fusion Using Kalman Filter

Kalman filter is a common method for sensor fusion because it can estimate parameters of a constantly changing system in real time, minimizing error covariance [121], although standard Kalman filter is not suitable for non-linear systems. Nowadays, several advanced Kalman filter methods are used for robotic systems, which were briefly mentioned before. For example, extended Kalman filter (EKF) is commonly used for non-linear systems [122]. First it constructs linear estimation, but then it is subsequentially updated. It is especially useful for merging sensor data with varying measurement models like GPS, IMU, and vision systems. Nevertheless, subsequential update of linear estimation requires calculating partial derivatives in each step, significantly increasing computational load. Unscanned Kalman filter (UKF) was created to work around the shortcomings of EKF, which can be applied for non-linear systems without direct laterization using sigma point approach for calculation mean and covariance. This method is very useful for accelerometer, GNSS and rotation sensordata fusion as presented in article [123].

Going further, cubature Kalman filter (CKF) was built upon its predecessors, which can deal with non-linear data with accuracy and reliability by performing high-dimensional state estimation. Nevertheless, it showed to suffer from error accumulation in long-term operations. In article [124], utilization of trifocal tensor geometry (TTG) for the CKF algorithm was suggested to increase filter estimation accuracy for long-term visual inertial odometry application.

Another recent filter showing great results for tracking large-scale moving objects is probabilistic Kalman filter (PKF). It simplifies conventional state variables, thus reducing computational load and making non-uniform modelling more effective. For example, in article [125], PKF-based non-uniform formulation is proposed for tackling escape problems in multi-object tracking and introducing a first fully GPU-based tracker paradigm. Non-uniform motion is modelled as uniform motion by transforming a time variable into a related displacement variable allowing to integrate deacceleration strategy into a control input model.

### 6.2. Sensor Fusion Using Particle Filter

It is another class of estimation algorithms that involves a probabilistic approach to estimate the state of the system. Particle filter (PF) stands out because of its ability to deal with non-linear system models and non-Gaussian noise. They also show great potential for localization and object detection tasks. For example, in article [126], PF is used for two ultrasonic sensors and radar fusion for a system that is able to navigate in unknown environments with static and dynamic obstacles. However, basic PF approaches are not suitable for real-time applications especially if the required number particles for accurate estimation is very high [127]. In article [128], an enhanced particle filter-weighted differential evolution (EPF-WDE) scheme is proposed, which is used to manage a non-linear and multidimensional system involving a variety of smartphone sensors with notable gains in accuracy and convergence.

### 6.3. Deep Learning for Sensor Fusion

Navigation systems are becoming increasingly complex with a large number of sensors with different physical nature. This amounts to large amount of imperfect raw data. Multi-modal sensor fusion architecture is essential in these cases, and deep learning (DP) techniques are emerging to tackle these tasks. DP is very effective because of non-linear mapping capabilities. Furthermore, DP models have deep layers that can generate high-dimensional representations, which are more comprehensive compared to previously mentioned methods. Also, it is very flexible and can be applied to a variety of applications [129]. In article [130], an adaptive-network-based fuzzy interface system (ANFIS) is proposed for LiDAR and inertial navigation system GNSS/INS fusion to localize indoor mobile robot. It incorporated human-like decision making with neural networks, which enables learning from data and improving performance, and it resulted in a lower standard deviation error compared to more classical EKF method. Another deep learning-based high-level fusion method for LiDAR and camera data is presented in article [131]. The author proposes high-order Attention Mechanism Fusion Networks (HAMFNs) for multi-scale learning and image expression analysis. It is capable of more accurate perception of surrounding the objects’ state, which is essential in autonomous driving.

## 7. Solution for Channel Robot Navigation System

Channel navigation is a special task due to restricted communication in working environments, complex layouts, lack of light, and unexpected obstacles. These problems are especially relevant for difficult-to-reach channels, which cannot be inspected in advance by the human eye. This kind of environment also requires focusing mainly on local navigation methods. Focusing on this scenario concept for obstacle detection, there is a robot localization and path planning procedure proposed. In addition, cost-effectiveness is taken into consideration.

### 7.1. Obstacle Detection

**Figure 4:**
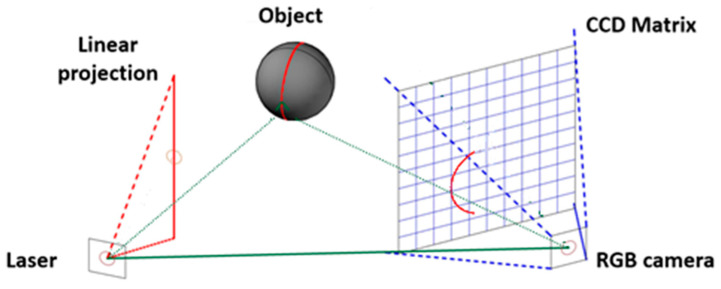
Obstacle scanning method using RGB camera and laser pointer.

Starting with obstacle detection, vision-based method were looked into. Obstacle detection in tight channels requires high accuracy and short-range detection. Depth camera and 3D time-of-flight sensors are a viable option because of their ability to evaluate distance to an object. Nevertheless, as discussed previously, implementation cost and computational resources are very high. For this reason, the combination of an RGB camera with a linear laser pointer was looked into. By using a modified laser triangulation method, it is relatively easy to detect an obstacle by focusing RGB and linear laser pointer to the same point and determining the change of linear laser projection in the presence of obstacles as shown in Figure 4.

**Figure 5:**
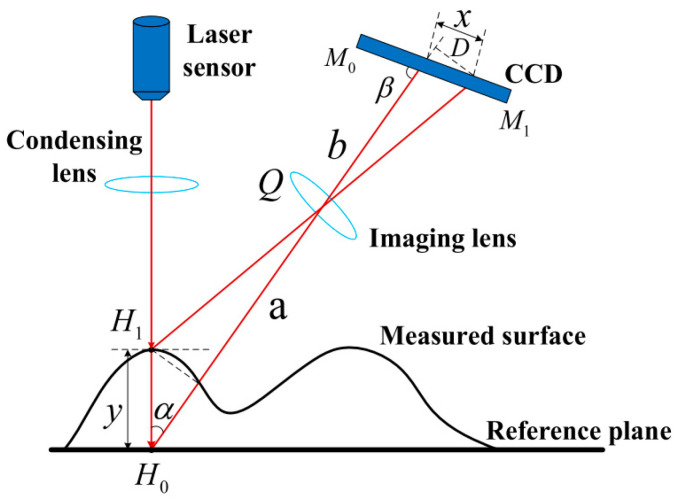
Laser triangulation method for displacement measurement [137].

The RGB camera image generated with CCD matrix can then be filtered to distinguish red color from the background, thus enabling to evaluate laser pointer projection as researched in articles [132,133,134,135], where this method was applied for obstacle avoidance. This method is also applied for object scanning as researched in articles [136], allowing to reach high accuracy. Projection will change according to the shape because the laser is pointed in an angle as shown in Figure 5.

**Figure 6:**
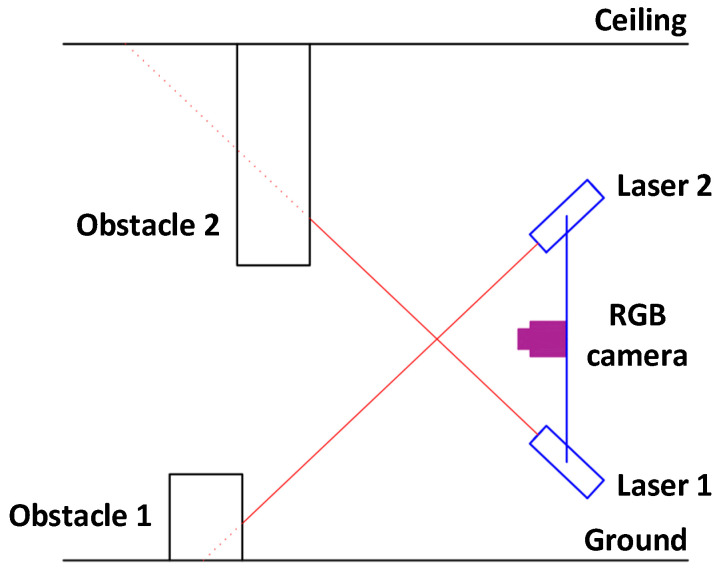
Ground- and ceiling-level obstacle detection.

Depending on the change in *y*, projection displacement *x* changes in CCD matrix. In article [138], two laser pointers were used for surface scanners because some smaller objects can be hiding behind bigger objects in front of the scanning system. One laser pointer can observe only one surface and obstacles with a base that starts from this surface. For instance, if one laser pointer is focused on the ground, it will not detect obstacles or detect them too late, then the base starts from the ceiling as shown in Figure 6.

**Figure 7:**
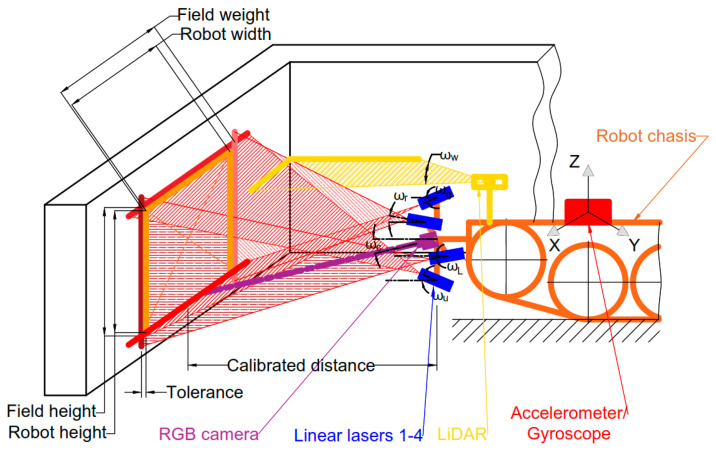
Channel robot navigation principal scheme.

This is an important factor to take into account when discussing navigation in the unknown channels because some unexpected obstacle bases can start from the ceiling or the wall. Configuration of the RGB camera and laser pointers was designed accordingly and is presented in Figure 7.

The configuration of four laser pointers was chosen to be able to detect obstacles from all four directions that could appear in the observed rectangle field. The size of the field is slightly bigger than robot geometry with fixed tolerance. The observed field should also be calibrated at a specific distance from the robot chassis, which depends on laser projection width and chosen angle. Moreover, pseudo-2D LiDAR of the fixed angle is added to widen the view of observation and to be able to double check information provided from the camera if an obstacle coincided with the observed plane. LiDAR sensor is also important for wall observation to perform wall-following navigations tasks, which will be explained further. Furthermore, accelerometers and gyroscopes are taken into consideration for relative and absolute rotation angle measurement. This allows performing correct motion then rotating robot chassis by having feedback from the sensors. Having multiple sensors allows increasing coverage and reliability of the system.

### 7.2. Sensor Fusion and Path Following Methodology

**Figure 8:**
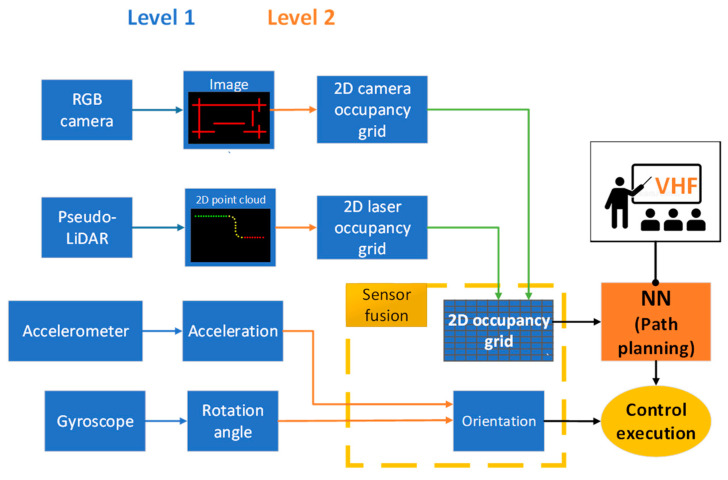
Sensor fusion and workflow of channel robot navigation system.

The next step for implementing the chosen sensors is to apply sensor fusion methods to obtain required data and optimize data processing. The main scheme of the sensor fusion and workflow of the system is shown in Figure 8 below.

**Figure 9:**
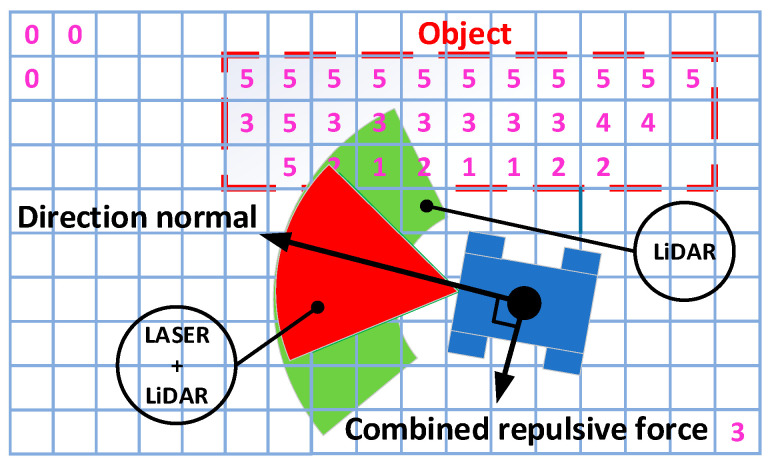
Modified wall following VHF-based path-planning scheme.

Sensors are interconnected using mid-level fusion to minimize computational resources required for directly processing raw data. This is of big importance for a cost-effective navigation system to maximize performance. Image data and 2D point cloud obtained from the RGB camera and LiDAR are converted into an occupancy grid expressed as one-dimensional arrays to simplify raw sensor data and make it easier to process it later using a path-planning algorithm. After that, camera and LiDAR arrays are fused into one 2D occupancy grid. Representation of an occupancy grid was selected because of the chosen vector field histogram (VFH) path planning algorithm because it is able to deal with complex obstacles and has good performance, although it has local minimum issues. The idea behind this method is that mobile robot working environment is converted into the grid and each cell represents a value of how close the object is from the robot as shown in Figure 9.

Then, according to the processed input data, obstacle values are combined to create an artificial vector, which represents repulsive force. According to the standard VHF algorithm, the repulsive force vector and attractive force vector are summed up to obtain a third vector, which represents moving direction as explained in articles [139,140]. Nevertheless, in our case, we have no global target position, and the system is purely local. For this reason, it is planned to adjust VHF method for wall following. Robot movement direction is determined by normal direction, which is placed 90 degrees from the combined repulsive force clockwise or counterclockwise depending on initial condition. Field of view is also separated into red and green regions, corresponding to combined laser and LiDAR field and solely LiDAR field, respectively. The idea is that obstacle avoidance initiates, then the obstacle intersects with the red field. On the other hand, the green field must always see an obstacle, and if that is not the case, must search for an obstacle. Due to this feature, the proposed system is able to follow the wall as a reference for the path and procced forward in the channel. In case of U-shaped obstacles, local minimum issues are reduced, although it is taken into account that maze-like channels will not have intersections and edges.

Channel robots as special cases for autonomous robot navigation is not the only solution for reviewed navigation systems. In general, development of robot navigation systems continues during ongoing robot technology, and recent findings in the review make a scratch for today’s situation.

## 8. Discussion and Conclusions

Going through the analyzed literature concerning mobile robot navigation, it is clear that hybrid sensor localization systems will be applied even more in the future. The combination of vision and distance sensors enhances the ability of object detection with accurate distance, color, and dynamic behavior estimation. Sensor hardware is also improving, in some cases creating modules combining several functions—for example, modules incorporating infrared, RGB, and ToF functionality. Furthermore, dynamic vision sensors (DVS) are rapidly improving with significant advantages over standard cameras with low latency, high dynamic range, and ability to estimate depth. Polarization filters also proved advantageous for vision technology by enhancing vision contrast and allowing detection of more difficult to perceive objects. Tactile sensor technology improves independently from optical navigation technologies. The soft structure of tactile sensors is able to inspect contact with obstacles with high accuracy, allowing navigation in very narrow spaces or even evaluating terrain properties and slippage.

Nevertheless, hardware technology advancement is relatively stable in comparison to software development, which will ring the main advantage in the future to enhanced performance of multi sensor systems. AI methods are proving to be effective in all stages of multi-sensor systems starting from post-processing individual sensor data to fusing and mapping the overall picture of the environment. For vision technology, new versions of YOLOv8 and YOLOv9 object detection systems are being built upon further for distinguishing small details in the image from large datasets. Deep learning architectures are progressively improving. CNN networks are commonly used for LiDAR and camera mapping. Nevertheless, transformer networks are being researched, which can increase performance of classification and mapping tasks especially when working with large datasets.

Advancement AI techniques for multi-sensor data processing, mapping, and path planning also allow use of cost-efficient sensors by enhancing software performance. The concept of a cost-efficient multi-sensor autonomous channel robot was presented incorporating a laser-RGB camera scanner, pseudo-LiDAR, and inertial sensors odometry. Future work will focus on incorporating deep learning methods for data fusion and path planning according to the research conducted in this survey.

## Figures and Tables

**Table 1:** Common mobile robot path planning methods.

Navigation G(Global), L(Local)	Method	Working Principle	Ref.
G	Dijkstra	Shortest path planning between established nodes	[10,11]
G	A star (A*)	Graphical search for shortest path to destination node	[12,13]
G	Artificial protentional field (APF)	Defined obstacles generate artificial repulsive force which in sum with attractive target force creates a path	[14]
G	Genetic algorithm	Heuristic methods that use mutation principle for optimal path generation from defined scenarios	[15]
G	Neural network (NN)	Learning algorithm that can be trained with known trajectory inputs and outputs to generate a path.	[16]
L	Bug	Moves in straight line to the target until obstacle is detected and evaded moving from one obstacle to another	[17]
L	Vector field histogram (VHF)	Occupancy grid is generated using sensor data, and target artificial force is attracting the robot while discrete obstacle data are pushing the robot; thus obstacles are evaded	[18]
L	Dynamic window	Sensor field of view is discretized in separate windows, which react to obstacles and maneuver to the target avoiding them	[19]
L	Fuzzy logic	Rule based method which can work with imprecise data using fuzzy values	[20]

**Table 2:** Non-vision-based robot localization technology review.

Sensor	Methodology	Path Planning Method	Advantages	Disadvantages	Ref.
LiDAR	2D LiDAR data are transformed in polar coordinates and clustering is performed using Euclidean algorithm	Improved time elastic band (TEB) method for local obstacle avoidance	System is able accurately react to dynamic obstacles, also by evaluating dynamic obstacle velocities, they can be evaded faster	Very dependent on localization algorithm accuracy	[30]
Tactile	Tactile sensors are used for target localization with robot hand setup	Path planning is realized using novel Virtual Tactile POMDP (VT-POMDP)-based method dedicated for partially observable domains	Allows to mimic human touch for object localization	Localization is not solved for scenarios with additional objects and obstacles	[31]
	Using tactile sensors, robot is able to react to obstacles and adjust trajectory to the goal	For global planning trajectory to the goal is estimated with A* algorithm	System is able to react not only to stationary but also to dynamic obstacles.	Field of view is low for obstacle detection, thus trajectory optimization is low. Contact is required for obstacle detection.	[32]
IR	Three coordinated IR distance sensors estimating distance to an obstacle.	Type 2 Fuzzy controller for local path planning	Good dynamic response and accuracy of the system	Very close objects cannot be detected also field of view is narrow	[33]
Ultrasonic	Ultrasonic sensors mounted on four sides of the robot for obstacle detection	Fuzzy controller is used for local path planning	Effective robot localization in simple maps	Not effective in very narrow spaces also influenced by sensors	[34]
Radar	A point cloud dataset called Milli noise captured with radar in indoor navigation scenarios	Dijkstra global path planning methods were used	Accurate point-wise labeling can be provided	High computational resources	[29]

**Table 3:** Non-vision robot localization technology comparison.

Criteria/ Method	Operating Distance	Field of View	Accuracy	Sensitivity to Disturbance	Computational Resources	Implementation Cost	Ref.
IR	10–400 cm	20–60°	2 mm	Moderate	Moderate	Moderate	[24,35]
Tactile	Contact	N/A	Up to 1%	Moderate	Low	Low	[36,37,38]
Ultrasonic	2 cm–10 m	15–30°	1–3 cm	Very high	Moderate	Low	[39,40,41]
Lidar	0.1–100 m	0–360° (3D, 2D)	1–3 cm (3D), <1 cm (2D)	High	Very High	High	[42,43,44]
Radar	1–300 m	120–360°	1–10 cm	Low	High	High	[45]

**Table 4:** Vision based robot localization technology review.

Sensor	Methodology	Path Planning Method	Advantages	Disadvantages	Ref.
RGB-D	Travers ability map is extracted from raw depth images using tiny-YOLOv3 to endure safe driving for low-body mobile robots	A* with fast marching method for faster distance cost calculation	Low-cost system enabling obstacle detection and path planning in real-time	Refresh rate is relatively slow for avoiding dynamically moving pedestrians	[55]
RGB-D	Closed-loop real-time dense RGB-D SLAM algorithm incorporating tiny-YOLOv4, which reconstructs dense 3D background for indoor mobile robot navigation	Optimal optimalRRT planner, which accelerates computation of faltered robot-centric point cloud for path planning	Provides faster computation of path planning in real time compared to conventional SLAM methods	Accuracy is affected by surface color, varying distance to the static, and dynamic objects	[56]
RGB	Pattern recognition using two laser pointers to detect and avoid obstacles using LaGrange interpolation formula to determine the distance	Rotation angle of the robot is adjusted according to the calculated distance of two laser pointers	Lower computation load and cost-efficient mobile robot navigation system	Influenced by light in a way that observed view does not have enough color contrast, also influenced by camera proximity	[57]
RGB	RGB CCD camera takes an image recognizing the red color shape drawn with laser pointer calculating velocity vector	Mobile robot performs steering tasks according to drawn shapes	Cost-efficient vision system enables to detect laser pointer trajectory	Precision of shape detection is affected by surface color and reflectivity	[58]
ToF	ToF camera is used for indoor obstacle detection where GPS signal is weaker then outside	Global path planning and local obstacle avoidance	Cost-efficient system for obstacle detection that is relatively accurate with different lighting of surfaces	In complex scenes, light can deflect multiple times, causing calculation problems	[59]
DVS	Multi-quadrotor localization and tracking is performed using event-based camera and deep learning network based on YOLOv5 and k-dimensional tree	MINLP-based motion planner, which enables quadrotor to calculate its position velocity and distance to other obstacles and quadrotors	Relatively cost-efficient system that is able to perform localization and path planning of multi-quadrotor systems	Limited field of view when object is close to camera, requires sufficient training data	[60]

**Table 5:** Vision-based robot localization technology comparison.

Criteria/ Method	Range	Color	Depth Accuracy	Sensitivity to Disturbance	Computational Resources	Implementation Cost	Ref.
RGB CCD	N/A	✔	N/A	High	Low	Moderate	[66,67]
RGB CMOS	N/A	✔	N/A	Very high	Low	Low	[68,69]
DVS	0.6–30 m	✔	61–98%	Low	Very high	Very high	[70,71]
RGB-D	0.5–10 m	✔	Up to 97%	High	High	High	[72,73,74]
ToF	0.35–10 m	N/A	Up to 99%	Moderate	Moderate	Moderate	[75,76,77]

**Table 6:** Hybrid robot localization technology review.

Sensor	Methodology	Path Planning Method	Advantages	Disadvantages	Ref.
2D LiDAR, RGB-D	Visual-based SLAM and laser-based Slam is used incorporating EKF based LiDAR and RGB-D fusion for environment mapping and robot localization	RRT* (Rapidly exploring random tree) global path planning and Fuzzy PID controller for following trajectory accurately	Integration of visual input provides richer data especially when LiDAR has a lack of data in wide areas	Additional visual maps increase computational load significantly	[80]
2D LiDAR RGB	Reinforced learning method uses visual data acquired with CenterNet depicting obstacles and projects these data in birds-eye view using LiDAR point cloud	While A* is implemented for global path planning, timed elastic bands (TEB) are implemented locally, complemented by reinforced learning	More accurate representation of distant and close objects using sensor fusion	High computational load, requires training data for image recognition and localization tasks	[81]
Radar, RGB	Visual data are inspected using Canny edge and then spatial fusion is used for camera and MMV radar to obtain data about the same target	Improved A* global method is used adding dynamic heuristic function for dynamic adjustment of cost between two points	Significantly improved object recognition and distance estimation for more accurate obstacle avoidance	Camera and radar require calibration for accurate data fusion result also sensitive to the distance to an object	[82]
Ultrasonic, RGB CMOS	YOLOv3 based on CNN is used to detect obstacles with camera, and fusion with ultrasonic sensor is used for distance estimation	Tested capability of obstacle detection for local navigations tasks	Allows to estimate distance to an object recognized by camera in real time	Accuracy of 90% for distance estimation, and recognition is relatively low	[83]
3D Lidar, DVS	Event camera compares changes in intensity for detection and acquired data is fused with point cloud by pairing clusters	Nonlinear Model Predictive Control (NMPC) is used for human tracking	Human detection is effective in high contrast zones	High computational resources	[84]
IR, Tactile, RGB	Fisheye camera to detect IR markers on soft skin structure is respective coordinate systems to detect tactile changes	Robot has three defined conditions including move towards the goal, move backward, and move along the object, which are controlled with PI controller	Highly accurate tactile data enabling to navigate in very narrow spaces	Complex calibration is required, friction with obstacles influences navigation accuracy	[85]

**Table 7:** Hybrid robot localization technology comparison.

Criteria/ Method	Range	Field of View	Accuracy	Sensitivity to Disturbance	Computational Resources	Implementation Cost	Ref.
Tactile/ RGB-D	0.5–10 m	60–180°	Up to 1%	Moderate	Moderate	Moderate	[88,89]
Ultrasonic/ RGB	2 cm–10 m	60–180°	1–3 cm	High	Moderate	Low	[90,91]
Lidar/RGB	0.1–100 m	360° (3D, 2D)	1–3 cm (3D), <1 cm (2D)	Very high	High	High	[92,93]
Lidar/ RGB-D	0.1–100 m	360° (3D, 2D)	1–3 cm (3D), <1 cm (2D)	High	Very high	Very high	[94,95]
Lidar/ DVS	0.1–100 m	360° (3D, 2D)	1–3 cm (3D), <1 cm (2D)	Low	Very high	Very high	[96,97]
Radar/RGB	1–300 m	120–360°	1–10 cm	Moderate	High	Moderate	[98,99]

**Table 8:** Low-level cooperative sensor fusion methods for mobile robot navigation.

Sensor	Methodology	Advantages	Disadvantages	Ref.
LiDAR, Camera	The point cloud generated using LiDAR is projected onto the image in real time. Point cloud is projected by colors referencing depth information	Accurate representation of the environment in real time for autonomous vehicle	High computational resources. Raw data has to be projected at a very high rate, faster than the acquisition rate	[105]
Stereo camera, LRF	Fusion-based human detection and tracking algorithm combining laser data-based search window and Kalman filter for recursive estimation of target position in robots coordinate system	Able to detect and track fast human movements in real time	High computational resources	[106]

**Table 9:** Mid-level cooperative sensor fusion methods for mobile robot navigation.

Sensor	Methodology	Advantages	Disadvantages	Ref.
Encoder, ultrasonic	Fuzzy logic algorithm input is acquired from ultrasonic sensors for obstacle detection and the motion is executed with feedback from wheel encoders	Fats and cost-efficient obstacle avoidance system	Slippage can introduce motion execution inaccuracies	[107]
3D LiDAR, GMSL camera	YOLACT image semantic segmentation algorithm for obstacle detection is used and then LiDAR point cloud is matched with the shape corresponding to camera pixels	Semantic segmentation algorithm allows for more accurate obstacle detection evaluating its shape	3D LiDAR requires high computational resources because of large point cloud	[108]
Encoder, compass	Odometry data are obtained based on wheels and encoders data and then fused with compass data using extended Kalman filter providing data for further movement to the target	Increased position accuracy to not more than 0.15 m	Inaccurate data are common because of wheel slippage, which cannot be evaluated by selected sensors	[109]
LiDAR, RGB camera	LiDAR and Camera joint calibration is initiated with spatial relationship, then target detection is initiated using deep learning PP-YOLOv2 and identification of surface using point cloud segmentation based on RANSAC.	Allows accurate detection of objects with suitable surfaces required for processing. Segmentation accuracy of 75.46%.	Suitable only for calibrated type of processed material. If material type changes, tuning is required.	[110]
IMU, LiDAR, RGB camera	A dense 3D map is obtained in real time using simultaneous localization and mapping (SLAM) while IMU sensors track short-term motion. Using reinforced learning RL and CNN algorithms obstacles are avoided mapping image and LiDAR data	Enables fully autonomous system allowing not only object recognition but also identification of various environmental factors	High computational resources. Requires training and large number of labeled data for ML algorithms	[81]
Global positioning system (GPS), IMU	GPS and IMU sensor data are interconnected using adaptive covariance matrix and adaptive unscented Kalman filter (AUKF) for vehicle position estimation	Outputs robust and accurate vehicle position estimation. AUKF yields better results than UKF or EKF filters	System is still affected by environment obstructions like buildings and depends on accurate kinematic model	[111]
Ultra-wideband (UWB), RGB-D	Kalman filter is used to reduce noise of UWB data, which are then fused with localization data acquired from image data processed with ORB-SLAM2. EKF is used for ORB-SLAM2/UWB fusion.	Significantly improves positioning accuracy compared to standalone UWB systems	Positioning accuracy strongly depends on field size because of UWB range limitations	[112]
3D LiDAR, RGB-D	For data fusion, first LiDAR and cameras are calibrated using edges for relative orientation parameters. Canny edge is used for extracting color image features. RANSAC is used for point cloud depth mapping. Extrinsic matrix is used for projecting point cloud onto an image.	Able to identify and locate cracks and evaluate geometric size with accuracy not more than 0.1mm using MobileNetV2-DeepLabV3	High computational resources for generating dense 3D point cloud and image semantic segmentation.	[113]
UWB, monocular camera	Histogram filter (RHF) is used for sensor fusion, which can handle exponential and Gaussian systems. Range information of UWB is fused with angle estimations received from the camera	66.67% reduction in angular error is achieved compared to standalone UWB systems	Positioning accuracy strongly depends on field size and anchor infrastructure because of UWB range limitations.	[114]
Gyroscope, accelerometer, magnetometer	Neural inertial tracking system (NeurIT) is incorporated, which incorporates RNN and transformers	Enables accurate indoor tracking, minimizing the drift appearing from extended periods or distances	System is only suitable for tracking	[115]

**Table 10:** High-level complementary sensor fusion methods for mobile robot navigation.

Sensor	Methodology	Advantages	Disadvantages	Ref.
Gyroscope, accelerometer, odometer, sonar	Unscented Kalman filter and Rauch–Tung–Striebel are applied to fuse raw Gyroscope, accelerometer, and odometer data for precise localization of the robot, then sonar point cloud is fused with sensor data for offset adjustment and environment calculations	Accurate robot localization and orientation acquisition.	3D environment representation. Sonar-based measurement introduces noise in closed environments.	[116]
UWB, encoder, speed sensor, accelerometer, GBSS, gyroscope	UWB and vehicle on board sensor fusion, which consisted of three components including multi sensor module, ARIMA-GARCH for UWB data processing, and global fusion module using AIMM and extended Kalman filter	Increases positioning accuracy in GNSS-challenging environments	Additional infrastructure for UWB is required, readability of communications must be ensured.	[117]
LiDAR, Camera, Radar	Image data are extracted using ResNet101-DCN, then dense 3D point cloud and sparse 3D point cloud are generated using VoxelNet, and then the postprocessed data of all sensors are merged using BEVFusion and SENet	Accurate and robust occupancy prediction of working environment even with challenging night and rainy scenarios	High computational resources for generating dense 3D point cloud	[118]

## Data Availability

No new data were created or analyzed in this study. Data sharing is not applicable to this article.
